# Cross-cultural adaptation and validation of the Finnish version of the central sensitization inventory and its relationship with dizziness and postural control

**DOI:** 10.1186/s12883-021-02151-6

**Published:** 2021-03-31

**Authors:** Jani Mikkonen, Hannu Luomajoki, Olavi Airaksinen, Randy Neblett, Tuomas Selander, Ville Leinonen

**Affiliations:** 1 Private practice, Helsinki, Finland; 2 Department of Surgery (incl. Physiatry), Institute of Clinical Medicine, University of Eastern Finland, Kuopio, Finland; 3 ZHAW School of Health Professions, Winterthur, Switzerland; 4 Department of Physical and Rehabilitation Medicine, Kuopio University Hospital, Kuopio, Finland; 5 PRIDE Research Foundation, Dallas, TX USA; 6 Science Service Center, Kuopio University Hospital, Kuopio, Finland; 7 Institute of Clinical Medicine-Neurosurgery, University of Eastern Finland, Kuopio, Finland; 8 Department of Neurosurgery, Kuopio University Hospital, Kuopio, Finland

**Keywords:** Central sensitization, Central sensitization inventory, Psychometric validation, Musculoskeletal pain, Chronic pain, Low back pain, Dizziness, Postural control

## Abstract

**Background:**

Central Sensitization (CS) involves dysfunction in neurophysiological mechanisms that increase neuronal responses to both noxious and non-noxious stimuli in the central nervous system. The Central Sensitization Inventory (CSI) is considered the leading patient-reported outcome measure for assessing CS-related symptoms. The aim of this study was to translate and cross-culturally adapt the CSI into Finnish (CSI-FI) and to evaluate its psychometric properties.

**Methods:**

Translation and cross-cultural validation of the CSI was conducted according to established guidelines. The validation sample was 229 subjects, including 42 pain free controls and 187 subjects with chronic musculoskeletal pain. The CSI-FI was evaluated for internal consistency, test-retest reliability, exploratory factor analysis with maximum likelihood extraction, relationship with subject-reported outcome measures [Tampa scale of kinesiophobia (TSK), the Depression scale (DEPS), 5-level EQ-5D version (EQ-5 L-5D), Roland-Morris Disability Questionnaire (RMDQ), and Pain and Sleep Questionnaire Three-Item Index (PSQ-3)], pain history, subjective symptoms of dizziness, and CS-related diagnoses on CSI part B. Furthermore, we studied the ability of the CSI-FI to distinguish pain free controls, subjects with chronic pain in a single body area, and subjects with multisite chronic pain. In addition, we studied the relationship of CSI-FI scores with postural control on a force plate.

**Results:**

The CSI-FI demonstrated good internal consistency (0.884) and excellent test-retest reliability (0.933) with a 7 ± 1 day gap between test administrations. Exploratory factor analysis with maximum likelihood extraction yielded a one factor solution. Fair to good correlations were found between the CSI-FI and the TSK, DEPS, EQ-5 L-5D, RMDQ, and PSQ-3. Subjective symptoms of dizziness correlated better with CSI-FI scores than any of the CS-related diagnoses on CSI part B. Total CSI-FI scores successfully distinguished between pain free controls, subjects with chronic pain in a single body area, and subjects with multisite chronic pain. The multisite pain group reported significantly more dizziness symptoms than the other two groups. Force plate measurements showed no relationship between postural control and CSI-FI scores.

**Conclusion:**

The CSI-FI translation was successfully cross-culturally adapted and validated into Finnish. CSI-FI psychometric properties and scores were all in acceptable levels and in line with previous CSI validations. The CSI-FI appears to be a valid and reliable instrument for assessing CS-related symptomology in Finnish-speaking populations.

## Background

Central sensitization (CS) involves dysfunction in neurophysiological mechanisms that increase neuronal responses to both noxious and non-noxious stimuli in the central nervous system [1]. It was originally defined by Woolf et al. 2011 as “an amplification of neural signalling within the central nervous system that elicits pain hypersensitivity” [2]. Underlying factors contributing to the CS phenomenon are complex, individualised, and not well understood [3].

The most common clinical signs and symptoms for CS-related pain syndromes include increased sensitivity to stimuli, lowered pain stimulus threshold, and prolonged pain after the stimulus has been removed [4]. Neurophysiological mechanisms of CS, with enhanced neuronal responses to stimuli, can contribute to prolonged symptoms in a wide variety of disorders. The term central sensitivity syndromes (CSSs) has been proposed to describe syndromes related to CS [5]. Pain is a predominant symptom in many CSSs, like fibromyalgia and irritable bowel syndrome. However, in some CSSs, like chronic fatigue syndrome or restless leg syndrome, pain is a secondary or non-significant symptom. CS can also contribute to trauma-related multidimensional symptomology, such as with whiplash syndrome [5, 6].

CS is one underlying multidimensional biopsychosocial factor leading to chronic pain [7]. Chronic musculoskeletal pain has been found to be associated with postural control instability compared to healthy controls [8, 9]. Postural control is a term used to describe regulation of the nervous system in order to produce and maintain a controlled, upright posture [10]. Instable postural control is one of the first signs and symptoms of many neurological diseases and cognitive impairments [11, 12] and is evident in fibromyalgia, which is a well-established CS-related pain syndrome [13, 14]. Subjective dizziness, vertigo and unsteadiness are also common symptoms of fibromyalgia [15–17].

The Central Sensitization Inventory (CSI) was developed in 2012 for clinical use to screen patients for symptoms related to CS and CSS [18]. The CSI is considered the leading patient-reported outcome measure (PROM) for assessing CS-related symptomology [19]. Although it has a short, less than ten year history, the CSI has been translated and validated in adult populations into many languages, including Dutch [20], Spanish [21], Brazilian Portuguese [22], Gurajati [23], Serbian [24], French [25], Japanese [26], Greek [27], Nepalese [28], Russian [29], and Italian [30]. Additionally, the CSI has been validated in an adolescent population in European Portuguese [31]. Published results from previous cultural adaptations and validations suggest that the CSI is a reliable, valid, and consistent measure [32]. Previous studies have shown good discriminative ability of the CSI to distinguish between chronic pain subjects and control subjects without pain [32–34]. Associations have been found between CSI scores and other validated patient-reported measures of CS-related symptoms, including depression symptoms, kinesiophobia, perceived disability, health-related quality of life, and sleep problems [26, 27, 30, 33]

Due to rationale linking chronic pain syndromes and fibromyalgia with postural control instability, it was hypothesized that there may be a potential relationship between higher CSI scores and postural control instability on a force plate. The force plate is mechanical sensing systems designed to measure the ground reaction forces and moments involved in postural control [36]. We also hypothesised that CSI scores may correlate with subjective dizziness symptoms. As far as we know, the relationships between CSI scores, postural control, and dizziness symptoms have not been researched previously.

The aim of this validation was to translate and cross-culturally adapt the CSI into Finnish and to evaluate its reliability (Internal consistency, test-retest reliability and measurement error), psychometric properties (relationship with other patient reported outcome measures), factor analysis (exploratory factor analysis with maximum likelihood extraction), and relationships between postural control on a force plate and subjective symptoms of dizziness. We also studied the discriminative ability of the CSI-FI with three subgroups: 1. Pain free controls, 2. Chronic pain in a single body area, and 3. Chronic multisite pain (pain in two or more body areas). This validation study adhered, where applicable, to the COonsensus-based Standards for the selection of health Measurement INstruments (COSMIN) checklist for methodological quality of studies on measurement properties of instruments [37].

## Methods

### Ethical approval and consent to participate

Ethical approval for the study was obtained from the Research Ethics Committee of the Northern Savo Hospital District with identification number 1106/13.02.00/2018. Written informed consent was obtained from all subjects prior to the study. The study was conducted in accordance with the declaration of Helsinki.

### Study subjects

**Fig. 1:**
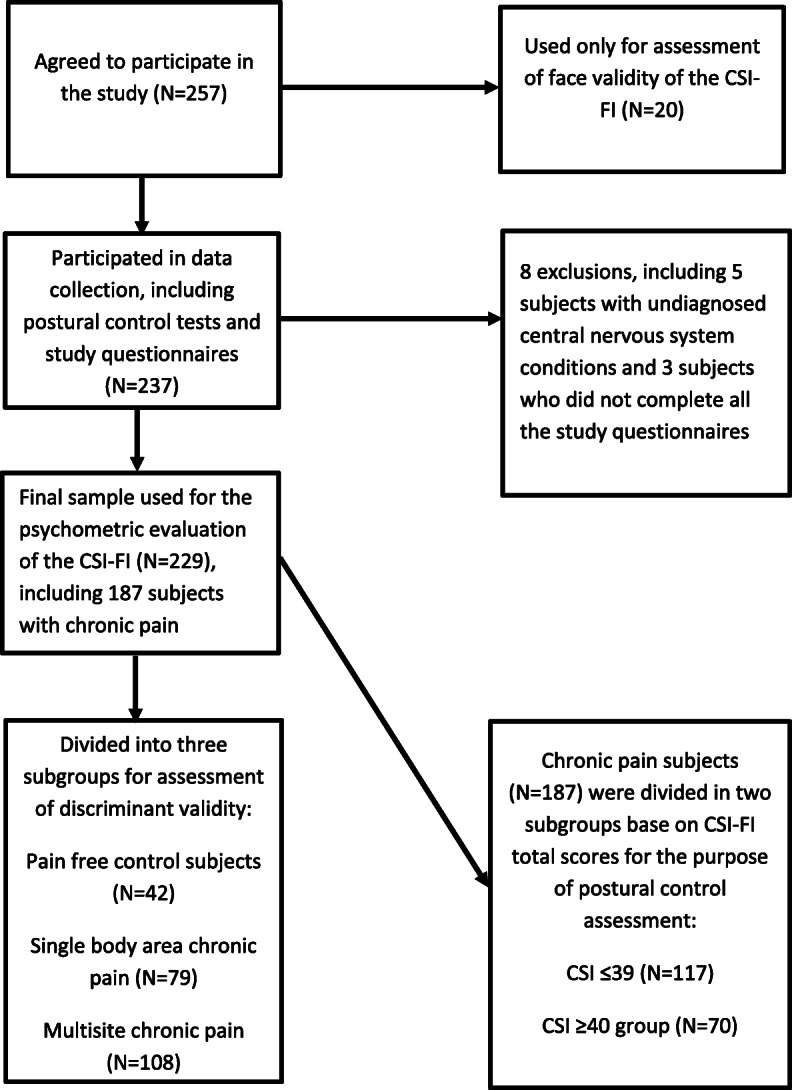
Flow chart of subjects

Study subjects were recruited through advertisements on the webpage of the Chiropractic private practice where the study was conducted and on other web pages and social media via different national Finnish musculoskeletal pain and spine-related organizations and healthcare colleagues. All 18–65 years old subjects meeting inclusion criteria for the study from the general population were invited to participate, whether they suffered pain or not. Inclusion criteria for the study were: 1) Age between 18 and 65 years 2) Proficient in written and spoken Finnish language. Exclusion criteria for the study were: 1) History of malignant tumour 2) History of diagnosed trauma or disease negatively affecting the central nervous system, including multiple sclerosis, Alzheimer’s disease, and dementia. A total of 257 subjects agreed to participate and booked clinical appointments through an online booking system. Three subjects were excluded because they did not complete the study questionnaires as instructed during the clinical appointments. Five additional subjects were excluded because of clear signs and symptoms indicating undiagnosed neurological pathological conditions affecting the central nervous system. After the exclusions, the total number of participants in the study was 249. Twenty of those participants were used only for feedback about the face validity of the Finnish translation of the CSI, and 229 subjects were used for the psychometric validation portion of the study. See the flow chart of subjects in Fig. 1.

### Translation and cross-cultural adaptation of the CSI

Translation and cross-cultural validation of the CSI from English into Finnish was conducted using standard guidelines, including a forward-backward translation method [35]. The multidisciplinary research group who authored this study included a chiropractor with post graduate qualifications in clinical neurology, Professor of Physiotherapy, Professor of Physical and Rehabilitation Medicine, licensed mental health professional and Professor of Neurosurgery. All these authors had more than ten years of clinical experience and expertise in chronic pain conditions and treatment.

First, permission to translate the CSI into Finnish was obtained from one of the original authors of CSI (author RN). After written permission was granted, the initial translation was made by the first author (JM) and a professional translator from English into Finnish. Both are native Finnish speakers and were blinded from each other’s translation. The first author has completed his undergraduate and post-graduate degrees in English speaking countries and the professional translator was a specialist in medical and related healthcare field texts.

Then second (HL) and last author (VL) formed an expert panel, independently reviewed the initial translations, chose the most appropriate translated items and provided comments and suggestions for changes to one item. All comments and suggestions were discussed with the first author. One translation problem was identified in item number 24 concerning childhood trauma. A direct Finnish translation of the option “always” was found to be obscure in the context of this item, so it was modified into a more appropriate Finnish word, which roughly translates to “frequently” in English. Also, some minor wording changes in content were made. A second professional translator and native English speaker, who was naïve to the CSI and purpose of this study, and who was also fluent in Finnish, created a backtranslation. The back translation was checked and approved by author RN, a native English-speaker.

Finally, the provisional CSI-FI was tested for face validity in twenty subjects. All subjects were informed about the details of the study and asked to give non-structured written or verbal feedback about their comprehension of each item on the questionnaire. All feedback about item comprehension was positive and all subjects were able to complete the CSI-FI without difficulty. Thus, the final version of the CSI-FI was established. Translated versions of the CSI, including the English and Finnish versions, can be found at https://www.pridedallas.com/questionnaires/.

### Data collection

Data were collected from May 2019 until March 2020 in a single Chiropractic private practice setting. The objective clinical measurements of postural control with a force plate and a structured interview assessing symptoms and history of dizziness during the past 12 months were collected during the clinic visit. After the clinic visit, subjects were asked to complete an online web-based battery of demographic information (age, gender, height, weight, and pain history) and clinical questionnaires at home, including the CSI-FI. Body mass index was calculated in the data analysis phase from subject-reported height and weight data. Subjects were then invited by email to complete the CSI-FI a second time 7 ± 1 days later for the purpose test-retest reliability assessment. The minimum number of subjects required for the test-retest evaluation was determined by previous CSI cultural adaptations and validations [18, 20, 26]. After one-hundred CSI-FI questionnaires were collected, no more email invitations were sent. All test-retest participants were asked to avoid starting any new types of pain medication and/or physical treatment, when ethically possible, during 7 ± 1-day gap between test administrations.

#### Subject-reported pain history questions

Each subject completed a structured web-based pain history, which asked dichotomous questions, including: chronic low back pain yes/no; referral to leg or leg pain yes/no (if yes to chronic low back); other chronic musculoskeletal pain yes/no; chronic headache yes/no; history of rheumatic disease diagnosed previously by physician yes/on. Subjects were also asked to rate pain severity on a numerical pain scale from 0 to 10 and to indicate pain duration in number of months with pain.

#### Subject-reported clinical outcome measures

The numerical pain rating scale (NPRS) is an eleven-point numerical scale. The scale is composed of 0 (no pain at all) to 10 (worst imaginable pain) and pain duration in months [38]. Chronic pain was defined as more than 3 months and more than 3 days per week.

The CSI is a two-part questionnaire [18]. Part A includes 25 questions about CS-related symptomology. The total score range is “0” to “100.” Higher scores indicate a higher number and frequency of CS-related symptoms. Frequency of symptoms is rated with the following in Likert scale options: 0 = never, 1 = rarely, 2 = sometimes, 3 = often, and 4 = always. Recommended clinical severity scores are: subclinical = 0 to 29; mild 30–39; moderate 40–49; severe = 50–59; and extreme = 60 to 100 [6, 39]. A recommended cut-off score for indicating clinically significant CS-related symptomology is 40 [6]. Part B asks with “No/Yes, and year diagnosed” about 7 possible previously diagnosed CSSs (Restless Leg Syndrome, Chronic Fatigue syndrome, Fibromyalgia, Temporomandibular Joint Disorder, Migraine or tension headaches, Irritable Bowel Syndrome, and Multiple Chemical Sensitivities) and 3 CS-related disorders (Neck injury including whiplash), Anxiety or panic attacks, and Depression). CSI B part is only for information and is not scored [6, 18, 39].

The Tampa scale of Kinesiophobia (TSK) is a 17-item questionnaire used to assess subjective kinesiophobia (fear of movement) [40]. The range of scores is from 17 to 68. Higher scores indicate an increasing degree of kinesiophobia. Items 4, 8, 12 and 16 are reverse-scored. Agreement with each item is rated with the following options: 1 = strongly disagree 2 = disagree 3 = agree 4 = strongly agree [40]. TSK has been translated into Finnish and validated in a Finnish population [40, 41].

The Depression Scale (DEPS) is widely used and well-validated 10-item questionnaire used to assess depressive symptoms. It has a score range between 0 and 30 [42]. Item responses are scored from 0 to 3 on four-point Likert scale options: 0 = not at all, 1 = a little, 2 = quite a lot and 3 = extremely. Higher scores indicate a higher possibility of a Major Depressive Disorder diagnosis. The DEPS has excellent structural validity for screening depressive symptoms among patients with low back pain [43].

Health related quality of life was measured with the Finnish translation of the 5-level EQ-5D version of the EuroQol (EQ-5D-5L) [44]. The questionnaire provides a simple descriptive profile of a respondent’s health status. The EQ-5D-5L questionnaire consists of five dimensions: mobility, self-care, usual activities, pain/discomfort, and anxiety / depression. Each dimension has five response levels: 0 = no problems, 1 = slight problems, 2 = moderate problems, 3 = severe problems, 4 = unable to /extreme problems. A second part of the EQ-5D-5L is the EQ visual analogue scale (EQ VAS). It records the respondent’s overall current health on a vertical visual analogue scale, where the endpoints are labelled ‘The best health you can imagine’ and ‘The worst health you can imagine’. The EQ-5D-5L and EQ VAS provide a quantitative measure of one’s overall health state [44]. EQ-5D-5L is scored from 0 = being dead to 1 = being in full health. An index value calculator was used to calculate a value between 0 and 1. Because there is currently no Finland standard value set available, a value set from Denmark was used to calculate the index value as recommended by the EuroQol EQ-5D-5L User Guide [45].

The Roland Morris Disability Questionnaire (RMDQ) is a well-validated 24-item questionnaire of disability in chronic low back pain populations [46]. The RMDQ is scored by adding up the number of items checked “yes” on different low back pain-related daily activity disabilities. Total scores range from 0 to 24, with higher scores indicating a higher level of disability related to low back pain [46, 47].

The Pain and Sleep Questionnaire Three-Item Index (PSQ-3) is a three-item sleep questionnaire designed to measure the impact of chronic pain on sleep during the past week [48]. The three questions are: “1. How often have you had trouble falling asleep because of pain?”, “2. How often have you been awakened by pain during the night?”,“3. How often have you been awakened by pain in the morning?”. These items were translated into Finnish for the present study. The original PSQ-3 used a visual analogue scale between 0 to 100 mm. However, we used a numerical eleven-point rating scale (NRS) from 0 to 10 in our version. This modification was made because of the difficulty in creating a visual analogue scale measurement in a universal digital format. In both versions, 0 indicates “never” and 100 mm or numerical scale 10 indicates “always.” Thus, the final score in our modified version ranged from 0 to 30 instead of 0–300.

#### Clinical tests of postural control on the force plate

Postural control was measured with a force plate. Because, the subjects completed the questionnaires after the clinic visit, the assessor was blinded from the participants’ pain histories and questionnaire scores. Postural control measurements included area and velocity of center of pressure (COP) displacement, which are the most commonly used parameters for postural control in chronic pain syndrome samples in previous studies [36, 49]. COP area and velocity describes the neuromuscular response to shifts in the body’s center of mass measured on the balance platform [50].

The postural control tests were carried out in the same room with identical conditions for each subject, including distance to the opposite wall and light on the room. COP displacement was assessed by a four-channel portable computerized force plate (BT4; HUR Labs Oy, Tampere, Finland). The force plate was calibrated prior to each individual’s measurement. Subjects were instructed with standardized instructions: to stand barefoot, feet as close together as comfortably as possible. If subjects found this foot stance unnatural, they were instructed to bring their feet further apart to create a more stable and natural-feeling standing stance. Subjects were instructed to look straight ahead and keep balancing in a relaxed manner. They were also instructed to keep their arms at their sides in a relaxed position. There was no clear fixation point for gaze, and the opposite wall was more than three metres away.

Four different postural control tests were carried out. Each test was 60 s, which was identical to the time measurement in previous studies on CLBP patients with a similar testing protocol [49]. A five second pre-phase period occurred before the actual COP measurement of 60 s. The sampling frequency was set to 50 Hz, which was recommended by the manufacturer to balance consistent data acquisition and manageable data size. The Hurlabs BT4 force plate has a sensitivity of 2,V/V + - 0.25% and an acceptable combined error maximum of 0.03%. Four COP displacement quiet stance measurements in respective order were: eyes open on a stable surface, eyes closed on a stable surface, eyes open on an unstable foam surface, eyes closed on an unstable foam surface. All measurements were carried out once, in similar (non-randomized) order, and there was no designated resting period between tests. A rectangular high density (50 kg/m3) closed-cell Airex Balance Pad (delivered by manufacturer with the force plate) was used for all tests requiring a foam surface in order to provide an unstable surface.

#### Subject subgroups

The 229 subject group was divided into different subsamples for various analyses. To test for postural control on the force plate, the chronic pain subjects (N = 187) were divided into two groups according to the recommended cut off score ≥ 40 for indicating clinically significant CS-related symptomology [6]. Of these 187 subjects, 117 (62.6%) scored below 40 and 70 (37.4%) scored above 40. Because we were only interested in assessing the relationship between CSI scores and postural control in subjects with chronic pain, pain-free control subjects were not included in this analysis.

To test for discriminative validity of the CSI-FI, the total subject sample was divided into three subgroups based upon self-reported pain symptoms, including pain free controls, single body area pain, and multisite chronic pain. Of the 229 total sample 42 (18.7%) reported no pain and were categorized as a control group. Specifically, the pain-free control subjects reported no CLBP, no radicular pain, pain scale 0/10, pain history 0 months, no other chronic musculoskeletal pain, and no chronic headaches. The rest of the sample (187; 81.3%) reported chronic pain. No subjects reported any acute or subacute pain. Of the 187 chronic pain subjects, 79 (34%) reported pain in a single body area (CLBP group with or without leg referral or other chronic musculoskeletal pain or chronic headache) and 108 (47%) reported multisite chronic pain (two or more of the following: CLBP with or without radiculopathy, other chronic musculoskeletal pain and/or chronic headache).

#### Subjective dizziness structured interview

A structured interview was done during the clinic visit about subjects’ dizziness history during the past 12 months. The questions for all subjects were:” Have you suffered dizziness during last 12 months? Dizziness means abnormal sensation causing disability for more than one day, which is not same as normal brief light headedness when standing up quickly”. Those subjects who reported dizziness which has resulted in any disability lasting more than one day (*n* = 53; 23%) were interviewed further about dizziness symptom subtypes. Dizziness subtypes were classified into seven classifications: 1. off balance or unsteadiness (*N* = 6), 2. light headedness (*N* = 12), 3. Feeling as if passing out (*N* = 8), 4. spinning or vertigo (*N =* 6), 5. floating or tilting sensation (*N* = 17), 6. blurring of vision when moving the head (*N* = 3) or other type (*N* = 0). This twelve-month time period of interest and symptom classification followed recent literature, which is based on symptoms and not actual diagnosis of any specific vestibular or neuro musculoskeletal condition [51].

## Statistical methods

Statistical analysis was performed using the SPSS version 25 (IBM SPSS Statistics for Windows, Version 25.0. Armonk, NY:IBM Corp.). Statistical significance was defined as *p* < 0.05. Data were shown as percentages or means with standard deviations. Group comparisons for normally distributed variables were executed by independent samples t-test or One-Way ANOVA model and for skewed distributed by Mann-Whitney U-test or Kruskall-Wallis test. Normality were checked by Shapiro-Wilks test. Categorical variables were compared by Chi-square or Fisher’s exact tests.

Cronbach’s Alpha was used to assess internal consistency. An Alpha value between 0.70 and 0.90 was considered good, and higher than 0.90 was considered excellent. Test-retest reliability was calculated by Intraclass correlation coefficient (ICC) from the second CSI-FI administration 7 ± 1 days later. ICC values ≤0.40 were considered to indicate fair reliability, 0.41–0.60 moderate reliability, 0.61–0.80 substantial reliability, and ≥ 0.81 excellent reliability. ICC with 95% Confidence Interval (CI) Lower-and Upper Bound were calculated for each item. Standard error of measurement (SEM) was calculated with formula: standard deviation*square root (1-ICC), where SD = standard deviation of change of CSI-FI scores from baseline to second administration.

A Kaiser-Meyer-Olkin measure and Bartlett’s Test of Sphericity were used to test for appropriateness of the factor analysis. Exploratory factor analysis (EFA) with maximum likelihood extraction were performed with the similar requirements for extraction as in a recent pooled multicountry CSI sample [19] The requirements for extraction were Kaiser criterion for components with Eigenvalues > 1.0 and the ratio of the Eigenvalue of the first and second unrotated component > 4.0. Inflection point on Cattel scree plot was used to observe similar finding on the graph [52, 53]. The item loading cut off value was set at 0.3. If the loading value was less than 0.3, the item was not retained as a factor.

Spearman’s correlation coefficients were used to investigate convergent validity of the CSI-FI by calculating the associations between total CSI-FI scores and scores on the NPRS, TSK, DEPS, EQ-5D-5L, RMDQ, PSQ-3 and dizziness history questions. Strengths of the associations were interpreted as: little or no correlation (< 0.25), fair correlation (0.25 > Rs ≤ 0.50), moderate to good correlation (0.50 > Rs2 ≤ 0.75), and good to excellent (Rs > 0.75).

## Results

There were no missing subject-reported data. Data collection with the online questionnaires reminded automatically if there were any missing items. Also, no subjects had a CSI-FI score of zero or of 100 so there were no data floor or ceiling effects.

### Total CSI-FI score distribution and demographic group differences between the chronic pain and pain-free control groups

The mean CSI-FI score in the total sample (*N* = 229) was 35.3 ± 12.2 with a CSI-FI score range from 11 to 70. The mean CSI-FI score in the pain-free control group (*N* = 42) was 28.0 ± 10.7 with CSI-FI score range from 12 to 43 and in the chronic pain group (*N* = 187) was 37.0 ± 12.0 with CSI-FI score range from 11 to 70. The total sample consistent of 67 males and 162 females. A statistically significant difference was found between the total CSI-FI scores in the chronic pain and control samples (*p* < 0.001). Mean age was also significantly different between these two subject groups (40.2 ± 10.6 years vs. 45.5 ± 11.8 years respectively; *p*-value .01). Other demographics of gender (male/female ratio; 54/133 vs. 13/29), height (171.2 ± 9.6 vs. 171.3 ± 8.2 cm), weight (75.1 ± 17.1 vs. 76.5 ± 15.9 kg), and body mass index (25.5 ± 4.9 vs. 26.0 ± 4.8 kg/m2) showed no significant differences between the two subject groups.

### Reliability

Internal consistency (Cronbach’s alpha) was good (0.884 and ICC 0.838; 95% CI 0.792–0.872) and test-retest reliability of Intraclass correlation coefficient (ICC) was excellent (0.933 and ICC 0.911; 95% CI 0.882–0.935). Standard error measurement (SEM) was calculated as 0.425.

Single item ICC was substantial-to-excellent on all items except item number six (I need help in performing my daily activities), which was moderate. Table 1. presents single item ICC and lower and upper bound confidence intervals (CI).

**Table 1:** Internal consistency – Cronbach’s alpha, intraclass correlation coefficient (ICC) 95% Confidence Interval (Lower-Upper Bound) of test-retest reliability

	Item	ICC	CI Lower-Upper Bound
1	I feel tired and unrefreshed when I wake from sleeping.	0.745	0.643–0.821
2	My muscles feel stiff and achy.	0.725	0.617–0.806
3	I have anxiety attacks.	0.794	0.708–0.856
4	I grind or clench my teeth.	0.895	0.848–0.928
5	I have problems with diarrhea and/or constipation.	0.759	0.661–0.831
6	I need help in performing my daily activities.	0.518	0.358–0.648
7	I am sensitive to bright lights.	0.739	0.635–0.817
8	I get tired very easily when I am physically active.	0.821	0.745–0.876
9	I feel pain all over my body.	0.761	0.664–0.832
10	I have headaches.	0.756	0.658–0.829
11	I feel discomfort in my bladder and/or burning when I urinate.	0.783	0.693–0.848
12	I do not sleep well.	0.795	0.709–0.857
13	I have difficulty concentrating.	0.723	0.614–0.805
14	I have skin problems such as dryness, itchiness or rashes.	0.849	0.784–0.896
15	Stress makes my physical symptoms get worse.	0.723	0.615–0.805
16	I feel sad or depressed.	0.763	0.667–0.834
17	I have low energy.	0.695	0.579–0.784
18	I have muscle tension in my neck and shoulders.	0.813	0.735–0.871
19	I have pain in my jaw.	0.868	0.810–0.909
20	Certain smells, such as perfumes, make me feel dizzy and nauseated.	0.860	0.799–0.904
21	I have to urinate frequently.	0.844	0.875–0.943
22	My legs feel uncomfortable and restless when I am trying to go to sleep at night.	0.819	0.743–0.875
23	I have difficulty remembering things.	0.815	0.736–0.871
24	I suffered trauma as a child.	0.897	0.851–0.929
25	I have pain in my pelvic area.	0.828	0.754–0.881

### Pain subgroup differences

The comparison of three pain subgroups on subject-reported clinical variables is presented in Table 2. On some variables, including the RMDQ and PSQ-3, the control group scored lowest, the localized pain group scored in the middle, and the multisite pain group scored the highest. The control group and localized pain group were statistically similarly but scored significantly lower than the multisite pain group on the CSI, EQ-5D-5L, and dizziness symptoms. Both pain groups were statistically similar, but scored higher on the TSK than the control group. In regards to pain-specific variables, the multisite pain group reported significantly more chronic low back pain, other musculoskeletal pain, and chronic headaches and longer pain duration than the localized pain group. There was no statistical difference in pain severity between the two pain groups.

**Table 2:** Subject-reported clinical variables among three subgroups (*N* = 229)

	Pain free control group (*N =* 42)		Chronic pain in a single body area (*N* = 79)		Multisite pain (two or more chronic pain locations) (*N* = 108)		*p-*value	Comparison between three groups
	Yes	No	Yes	No	Yes	No	χ2	χ2
Chronic low back pain	0 (0%)	42 (100%)	56 (71%)	23 (29%)	105 (97%)	3 (3%)	< 0.001*	a (< 0.001*) b (< 0.001*) c (< 0.001*)
Referral to leg or leg pain (if chronic low back pain was indicated)	0 (0%)	42 (100%)	28 (35%)	51 (65%)	59 (55%)	49 (45%)	< 0.001*	a (< 0.001*) b (< 0.001*)
Other chronic musculoskeletal pain	0 (0%)	42 (100%)	23 (29%)	56 (71%)	106 (98%)	2 (2%)	< 0.001*	a (< 0.001*) b (< 0.001*) c (< 0.001*)
Subject reported Chronic headache	0 (0%)	42 (100%)	0 (0%)	71 (100%)	24 (22%)	84 (78%)	< 0.001*	b (< 0.001*) c (< 0.001*)
Previous Rheumatic disease diagnosis	0 (0%)	42 (100%)	2 (2%)	77 (98%)	8 (7%)	100 (93%)	0.84	
Dizziness during last 12 months	4 (10%)	38 (90%)	9 (11%)	70 (89%)	40 (37%)	68 (63%)	< 0.001*	b (< 0.001*) c (< 0.001*)
							One-Way ANOVA	Post hoc LSD
Numerical pain scale	0 ± 0		3.8 ± 2.8		4.7 ± 2.1		< 0.001*	a (< 0.001*) b (< 0.001*)
Pain in months	0 ± 0		39.7 ± 6.9		76.7 ± 83.7		< 0.001*	a (0.04*) b (< 0.001*) c (< 0.001*)
CSI score	28.0 ± 10.7		31.8 ± 10.1		40.7 ± 11.7		< 0.001*	b (< 0.001*) c (< 0.001*)
TSK	25.8 ± 4.9		30.2 ± 6.8		33.0 ± 7.7		< 0.001*	a (0.01*) b (< 0.001*)
DEPS	5.4 ± 4.3		4.8 ± 3.9		7.3 ± 5.4		0.01*	c (< 0.001*)
RMDQ	0.4 ± 0.9		2.6 ± 3.1		4.4 ± 3.7		< 0.001*	a (< 0.001*) b (< 0.001*) c (< 0.001*)
PSQ-3	2.0 ± 3.6		6.2 ± 6.6		9.3 ± 7.4		< 0.001*	a (0.01*) b (< 0.001*) c (0.01*)
EQ-5D-5L	0.85 ± 0.097		0.79 ± 0.079		0.74 ± 0.129		0.01*	b (< 0.001*) c (0.01*)

One-Way ANOVA post hoc comparison of Fisher’s Least Significant Difference (LSD). Statistical significance *p <* 0.05*. Comparison between control group without pain and single body area pain (a), between control group without pain and multisite pain (b), and between single body area pain and multisite pain (c)

### Number of subject-reported diagnoses on CSI part B

As shown in Table 3, the total number of CS-related diagnoses reported in the control group was statistically different among the three subject groups. Though there was a clear trend for the multi-site pain group to report the most individual CSSs and the control group to report the least, only irritable bowel syndrome was statistically significant. The most prevalent CSS diagnosis reported in our sample was depression (*N* = 56).

**Table 3:** Number of subject-reported diagnoses on CSI part B by three subject subgroups (*N =* 229)

CSS diagnoses	N	Control group without pain (*N =* 42)	Single body area pain (*N =* 79)	Multisite pain (*N =* 108)	Pearson Chi-Square (χ2)	Comparison between groups (χ2)
1 Restless Leg Syndrome	5	0	3 (60%)	2 (40%)	0.376	
2 Chronic Fatigue Syndrome	1	0	0	1 (100%)	0.570	
3 Fibromyalgia	5	0	2 (40%)	3 (60%)	0.560	
4 Temporomandibular Joint Disorder (TMJ)	32	6 (18.8%)	7 (21.9%)	19 (59.4%)	0.235	
5 Migraine or tension headaches	35	3 (8.6%)	12 (34.3%)	20 (57.1%)	0.221	
6 Irritable Bowel Syndrome	35	1 (2.9%)	6 (17.1%)	28 (80%)	<0.001*	b (0.01*) c (0.01*)
7 Multiple Chemical Sensitivities	7	1 (14.3%)	1 (14.3%)	5 (71.4%)	0.402	
8 Neck Injury (including whiplash)	17	0 (0%)	6 (34.5%)	11 (64.7%)	0.102	
9 Anxiety or Panic Attacks	29	6 (20.7%)	7 (24.1%)	16 (55.2%)	0.453	
10 Depression	56	5 (8.9%)	17 (30.4%)	34 (60.7%)	0.330	
					Kruskal- Wallis Test	Mann-Whitney Test
Total number of CSS diagnoses	222	23 (10.4%)	61 (27.5%)	138 (62.1%)	<0.001*	b (<0.001*) c (0.005*)

Pearson Chi-Square (χ2), statistical significance *p <* 0.05*. Comparison between control group without pain and single body area pain (a), between control group without pain and multisite pain (b), and between single body area pain and multisite pain (c). Total number of CSS diagnoses Kruskal-Wallis Test and Mann-Whitney Test, statistical significance *p <* 0.05*

### Factor analysis

**Fig. 2:**
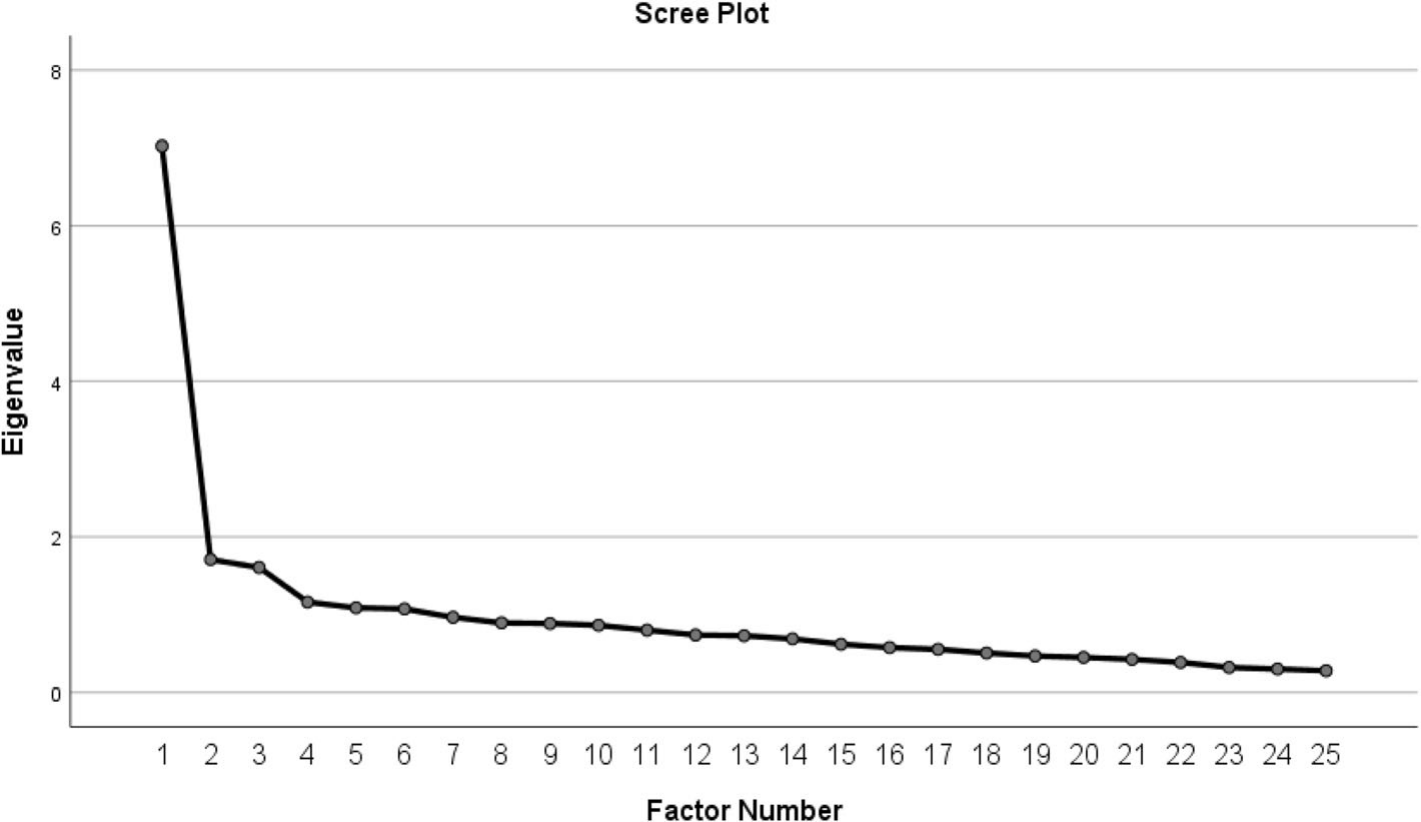
Scree plot of factor analysis

Values of the Kaiser-Meyer-Olkin measure (0.870) and Bartlett’s Test pf Sphericity (*p* < 0.001) indicated that a factor analysis was appropriate for this study sample. The exploratory factor analysis (EFA) factor 1 explained 28.1% (Eigenvalue 7.026) and factor 2 explained 34.9% (1.706) of the total variance. A scree plot with eigenvalues is shown in Fig. 2.

Results of the Exploratory Factor Analysis are presented in Table 4. The ratio of factor 1 Eigenvalue (7.026) and factor 2 Eigenvalue (1.706) were 4.12, which met ≥4 requirement for a one factor model [52]. All items had a required factor loading ≥0.3. Additionally, recommendation for suitability for factor analysis of at least five subjects per item were met [53, 54]. A total of 229 subjects and 25 items added up to 9.2 subjects per item. Factor loading descriptive statistics and factor loading for each question are presented on the Table 4.

**Table 4:** Exploratory Factor Analysis one factor model

	CSI Items	Mean	SD	Factor loading
1	I feel tired and unrefreshed when I wake from sleeping.	2.24	.84	.50
2	My muscles feel stiff and achy.	2.38	.83	.49
3	I have anxiety attacks.	1.03	.94	.58
4	I grind or clench my teeth.	1.19	1.12	.32
5	I have problems with diarrhea and/or constipation.	1.51	.93	.43
6	I need help in performing my daily activities.	.25	.53	.43
7	I am sensitive to bright lights.	1.24	1.16	.46
8	I get tired very easily when I am physically active.	1.38	1.00	.60
9	I feel pain all over my body.	.75	.94	.56
10	I have headaches.	1.42	.81	.50
11	I feel discomfort in my bladder and/or burning when I urinate.	0.36	.70	.40
12	I do not sleep well.	1.98	0.92	.50
13	I have difficulty concentrating.	1.66	.83	.58
14	I have skin problems such as dryness, itchiness,or rashes.	1.52	1.07	.48
15	Stress makes my physical symptoms get worse.	2.14	1.06	.64
16	I feel sad or depressed.	1.42	.79	.56
17	I have low energy.	1.88	.77	.64
18	I have muscle tension in my neck and shoulders.	2.36	.95	.60
19	I have pain in my jaw.	.88	1.01	.53
20	Certain smells, such as perfumes, make me feel dizzy and nauseated.	.97	1.17	.49
21	I have to urinate frequently.	1.54	1.14	.43
22	My legs feel uncomfortable and restless when I am trying to go to sleep at night.	.93	0.99	.42
23	I have difficulty remembering things.	1.51	.86	.52
24	I suffered trauma as a child.	.96	1.16	.42
25	I have pain in my pelvic area.	2.10	1.18	.30

### Convergent validity of the CSI-FI

As shown in Table 5, fair to good correlations were found between the CSI-FI and the Tampa scale of kinesiophobia, the Depression Scale, 5-level EQ-5D version, Roland-Morris Disability Questionnaire, and Pain and Sleep Questionnaire Three-Item Index.

**Table 5:** Correlations between total CSI-FI scores and subject-reported outcome measures and pain and dizziness history (*n =* 229)

Clinical Variables	Correlation with CSI-FI
**Subject-reported outcome measures**	
Tampa Scale of Kinesiophobia (TSK)	0.463*
Depression Scale (DEPS)	0.615**
The Roland-Morris Disability Questionnaire (RMDQ)	0.387*
The Pain and Sleep Questionnaire Three-Item Index (PSQ-3),	0.505**
EuroQol The 5-level EQ-5D version (EQ-5D-5L)	−0.554**
**Pain and dizziness history**	
Chronic low back pain	0.245
Pain referral to leg	0.291*
Other chronic musculoskeletal pain	0.425*
Numerical Pain Rating Scale (NPRS)	0.290*
Pain duration in months	0.253*
Chronic headache	0.255*
Dizziness in the last 12 months	0.323*
**CSS Number of subject-reported diagnoses on CSI part B**	
1 Restless Leg Syndrome	0.055
2 Chronic Fatigue Syndrome	0.101
3 Fibromyalgia	0.142
4 Temporomandibular Joint Disorder (TMJ)	0.223
5 Migraine or tension headaches	0.295*
6 Irritable Bowel Syndrome	0.296*
7 Multiple Chemical Sensitivities	0.163
8 Neck Injury (including whiplash)	0.066
9 Anxiety or Panic Attacks	0.163
10 Depression	0.305*

Little or no correlation (<0.25), Fair correlation (0.25 > Rs ≤ 0.50) *, moderate to good correlation (0.50 > Rs2 ≤ 0.75) **

Small correlations were found between the CSI-FI score and chronic low back pain. Fair correlations were found between CSI-FI and pain referral to the leg, other chronic musculoskeletal pain, numerical pain rating scale, pain duration, chronic headache and dizziness during last 12 months.

Small correlations were found between the CSI-FI and subject-reported fibromyalgia and temporomandibular joint disorder, and fair correlations were found with migraine or tension type headaches, irritable bowel syndrome and depression.

### CFI-FI and postural control

Subgroup comparisons among the chronic pain subjects by CSI-FI cut off scores ≥40 on postural control parameters are presented in Table 6. No significant differences were found between the two subject groups in age, gender, height, weight, or body mass index. No statistical differences were measured between the subject groups in the eight clinical postural tests, although the “Eyes closed on unstable surface velocity of COP” test approached significance.

**Table 6:** Postural control on force plate on groups of recommended CSI cut off score ≥ 40 on subjects with chronic pain (*n =* 187)

Test on force plate	CSI ≤39 group (*N* = 117) mean ± SD	CSI ≥40 group (*N* = 70) mean ± SD	*p-* value
Eyes open on firm surface area of COP [mm^2^]	296.4 ± 164.0	274 ± 161.8	0.32
Eyes open on firm surface velocity of COP [mm/s]	9.7 ± 3.0	9.3 ± 2.8	0.40
Eyes closed on firm surface area of COP [mm^2^]	464.1 ± 356.8	433.2 ± 362.7	0.28
Eyes closed on firm surface velocity of COP [mm/s]	15.8 ± 7.7	14.5 ± 5.7	0.31
Eyes open on unstable surface area of COP [mm^2^]	510.0 ± 251.0	466.4 ± 244.0	0.24
Eyes open on unstable surface velocity of COP [mm/s]	14.6 ± 4.3	14.1 ± 3.6	0.54
Eyes closed on unstable surface area of COP [mm^2^]	1142.2 ± 641.0	1028.6 ± 544.4	0.20
Eyes closed on unstable surface velocity of COP [mm/s]	31.0 ± 11.4	28.0 ± 9.8	0.06

Mann-Whitney Test, statistical significance *p <* 0.05*

## Discussion

Comparing CSI scores across study populations is very difficult due to the considerable heterogeneity of the different validation cohorts and study reporting practices. A recent study in Spain, with a similar chronic musculoskeletal pain cohort as ours, found a lower mean total CSI score of 24.6 compared to 37.0 in our sample [55]. The Spanish study did not separate subjects with single and multiple pain sites and had considerably fewer females (44.3%) than our study (71.7%), which might explain to some extent their lower mean total CSI score. Multiple pain sites is one of the main clinical hallmarks of the central sensitization and female gender is one the main risk factors for development of central sensitization-related chronic pain [1–3]. In other validation studies, CSI scores with adult chronic pain populations have ranged between 21.9. and 52.4 [18, 20, 24, 26–28, 30]. Cultural differences in symptom-reporting, differences in the clinical make-up of the study samples (e.g. gender ratios, chronicity of different pain syndromes) and reporting practices of different studies (e.g. different methods of subgrouping subjects), health history comorbidities (e.g. mental disorders, previous injuries affecting the central nervous system), and even genetic differences in different country populations might help explain the variation in total CSI scores.

The CSI-FI reliability (Internal consistency = 0.884 and test-retest reliability = 0.933) were very similar to previous validations, where the range of Cronbach’s alpha has varied from 0.88 to 0.99 and the range of test-retest has varied from 0.85 to 0.99 [27, 28, 32]. Though this was the first CSI-validation which used a digital data collection format, we found similar reliability results to previous traditional paper format data collection formats. Test-retest ICC lower and upper bound were excellent, in spite of CSI question 6 (I need help in performing my daily activities), which showed somewhat lower substantial reliability.

SEM values assess the likelihood of a “true” score, which represent a reliable score without any fluctuations from systematic and random factors related to the measurement process. The general rule for interpreting results is that lower scores indicate higher reliability and more confidence that the score has been measured accurately. In the present study, SEM was 0.42, which indicated that the CSI-FI scores were close to the “true” score. In previous validations, only the Nepali CSI reported a smaller SEM (0.31) score [28]. In other CSI validations, SEM scores have ranged from 1.84 to 3.16 [21, 23, 24, 27].

A previous factor analysis (EFA with maximum likelihood extraction) in a large pooled multicountry sample yielded a bifactor solution, in which one general “CS-related symptoms” factor showing “substantial reliability.” Four subfactors were clearly identified, but their reliability was not sufficient enough to be recommended for use as individual subscales in research or clinical work [8]. Because of this recommendation, CSI subscales were not used in the present study. Our data met the same requirements as the multicountry study, although our factor loading was set at 0.3 instead of 0.4. Two items in our sample, 4 and 25, loaded between 0.3 and 0.4, so the correlation coefficients were somewhat weaker compared to the multicountry sample. Additionally, in our sample, factor 1 explained 28.1% of the variance, whereas in multinational sample, factor 1 explained 36.1% of the variance. Hence, the percentage of explained variance was somewhat lower in our study. However, in previous Spanish [21] and Italian [30] validations, which also yielded 1 factor model solutions, factor 1 explained only 25.9 and 26% of the variance, respectively, which are lower than in the present validation. It should be noted that the multicountry CSI study had a much larger study cohort (*n* = 1987) and more heterogeneous sample of subjects than other validation studies, including ours, which may explain some differences in the results.

Fair correlations (0.25 > Rs ≤ 0.50) were found between the CSI-FI and other subject-reported variables, including the TSK (kinesiophobia), RMDQ (disability) pain history questions of pain referral to leg, other chronic musculoskeletal pain, numerical pain rating scale, pain duration in months, chronic headache, and dizziness in last 12 months. Moderate-to-good correlations were found between the CSI-FI and the DEPS (depression), PSQ-3 (impact of pain on sleep) (0.50 > Rs2 ≤ 0.75) and negatively moderately-to-good correlations were found with the EQ-5D-5L (quality of life). The correlation between CSI-FI scores and subject-reported chronic low back pain was not significant. Pain history findings are difficult to compare to previous validations because of the heterogeneity of study samples, data collection methods, and collection of clinical variables that are not standardised as in PROMs. In one previous validation, the TSK was studied in an adolescent population [31] and EQ-5D-5L [26] and RMDQ [30] in an adult population [26], which showed similar correlations with the CSI. The relationship between the CSI-FI and the patient-reported outcome measures in the present study provide additional evidence of the convergent validity of the CSI.

The total subject sample was divided into 3 subject groups for discriminative analysis, including a pain-free group, localized chronic pain group, and multi-site chronic pain group. It was assumed that the multisite chronic pain group would likely have the most CS and the control group the least amount of CS among the groups. In fact, the multisite pain group reported the highest number of CSSs, and the control group reported the lowest number of CSSs, on CSI part B. In addition to more widespread pain, the multi-site pain group reported significantly more perceived level of disability, depressive symptoms, perceived level of disability, poorer quality of life, pain-related sleep problems, chronic headaches, and longer pain duration than the localized pain group. Compared to the localized pain group, the multisite group scored significantly higher on the CSI-FI. Associations have been found between CSI scores and similar symptom reporting in other previous studies [27, 33, 34]. The results of the present study provide further evidence of discriminant validity of the CSI.

The relationship between CSI scores and subjective symptoms of dizziness has not been studied previously. Dizziness is a common symptom with many possible underlying causes, but it is not an actual diagnosis pointing into one specific cause of dizziness [56]. Subjective dizziness symptoms were divided in six different subtypes in the present study, pointing to different possible proprioceptive, visual, and/or vestibular dizziness diagnoses. Interestingly, significantly more patients in our multi-site chronic pain group, which is very well-known hallmarks of central sensitivity [1, 2] reported dizziness (37%) comparing to both the localized chronic pain group (11%) and the pain-free control group (10%). Furthermore, dizziness correlated better with the CSI-FI than most of the pain symptoms (chronic low back pain, pain referral to leg, numerical pain rating scale, pain duration in months and chronic headache) or any of the CSS diagnoses on CSI part B. It was known from previous studies that dizziness is a common symptom in patients with a fibromyalgia diagnosis [15–17]. We were unable to fully assess the relationship between dizziness and fibromyalgia in the present study population because only five subjects had a fibromyalgia diagnosis. However, the relatively high prevalence of dizziness symptom in our multisite pain group, and the relationship between dizziness symptoms and total CSI-FI scores, suggests that further research on this topic is warranted.

Some previous studies (but not all) have demonstrated associations between CSI scores and objective measures of central sensitization, including chemical measurements of brain derived neurotrophic factor [22], gamma aminobutyric levels [57], and a cold-pressure conditioned pain modulation test [22]. We hypothesized a relationship between higher CSI scores and poorer postural control test results on a force plate. Previous studies have shown differences in postural control between patients with a fibromyalgia diagnosis and pain-free controls, especially when visual information was missing [58]. We assessed COP and velocity COP because they have been the most commonly used parameters in previous research on low back pain populations [36, 49]. The postural stability testing was carried out with two groups with significantly different CSI scores, but no significant demographic differences on age, gender, height, weight or body mass index, and an appropriate and detailed test protocol with an adequate sample size. Two out of four tests were carried out with eyes closed to assess the impact of missing visual information. However, our study failed to show a relationship between higher CSI-FI scores and poorer postural control.

### Strengths and limitations

#### Strengths

Some strengths of this study included a very thorough validation of different measurement properties of the CSI-FI, including cross-cultural validity, face validity, internal consistency, test-retest reliability, measurement error, discriminant validity, structural validity, and convergent validity with an adequate subject cohort size. Furthermore, two novel measurements were assessed in relation to CSI-FI scores, including state-of-the-art postural control testing and subjective symptoms of dizziness.

#### Limitations

As with other studies of this kind, the results are based on one subject sample in a single clinic, so generalization to other subject populations should be made with caution. All symptoms were self-reported, and pain reporting was limited by the items on a single questionnaire. Actual medical diagnoses by a trained clinician were lacking. However, the self-reported data from our subject sample showed no discrepancies or illogical patterns of answers to suggest that they were invalid or had any considerable negative effects on our findings. Furthermore, it can also be noted that musculoskeletal pain symptoms and clinical diagnoses were primarily determined by  subject self-report and not by trained clinicians [59]. The study advertisement specified “force plate measurements, personal feedback, and standardised exercises,” so we might have attracted subjects with balance problems, which may have explained a surprisingly high proportion of subject-reported dizziness symptoms. The study clinic also specializes in treatment of balance impairments, which might have encouraged more subjects with balance problems and dizziness to volunteer for the study. Hypothesis testing was not carried out, which is recommended on the COSMIN checklist because we felt it would not add any validity to the successful validation of the CSI-FI.

#### Suggestions for further research

CSI part B is limited to 7 CSSs and 3 related diagnoses. Additional CSSs and related symptoms (such as dizziness) could be added to CSI part B data collection and studied along with total CSI scores. This might help clinical symptom-based sub-classification of different CSS syndromes and hence developing more specific treatment for different CSS syndromes. Based on the results of the current study, additional research on the relationship of dizziness and CSI scores is warranted. Possibly subtype of CS-related dizziness and targeted treatment protocols for dizziness could be developed.

The wide range of average CSI mean scores across different CSI validation studies needs further elaboration - maybe a review-type study comparing similarities and differences of different CSI validations in different subject populations.

## Conclusions

The CSI was successfully cross-culturally adapted and validated into Finnish. The psychometric properties of internal consistency, test-retest reliability, measurement error, content validity, convergent validity, and discriminative validity were all found to be acceptable and in line with previous successful CSI validations. The CSI-FI may be a useful screening tool for assessing CS/CSS-related symptoms in clinical and research populations among Finnish speaking patients.

## Data Availability

The datasets used and/or analysed during the current study available from the corresponding author on reasonable request.

## Abbreviations

CS: Central Sensitization
CSI: Central Sensitization Inventory
CSI-FI: Central Sensitization Inventory Finnish
TSK: Tampa Scale of Kinesiophobia
DEPS: The Depression Scale
EQ-5 L-5D: 5-level EQ-5D version
RMDQ: Roland-Morris Disability Questionnaire
PSQ-3: Pain and Sleep Questionnaire Three-Item Index
CSSs: Central Sensitivity Syndromes
COSMIN: The COnsensus-based Standards for the selection of health Measurement INstruments
NPRS: The numerical pain rating scale
COP: Center of pressure
ICC: Intraclass correlation coefficient
CI: Confidence Interval
SEM: Standard error of measurement
EFA: Exploratory factor analysis
Rs: The Spearman’s rank correlation coefficient
